# Imaging Lymphatic System in Breast Cancer Patients with Magnetic Resonance Lymphangiography

**DOI:** 10.1371/journal.pone.0069701

**Published:** 2013-07-05

**Authors:** Qing Lu, Jia Hua, Mohammad M. Kassir, Zachary Delproposto, Yongming Dai, Jingyi Sun, Mark Haacke, Jiani Hu

**Affiliations:** 1 Department of Radiology, Shanghai Renji Hospital, Shanghai Jiao Tong University School of Medicine, Shanghai, China,; 2 Department of Radiology, Wayne State University; Michigan, United States of America,; 3 Department of Radiology, Henry Ford Hospital, Detroit, Michigan, United States of America; 4 MR Business, Greater China, Philips Healthcare, Shanghai, China; The Norwegian University of Science and Technology (NTNU), Norway

## Abstract

**Objective:**

To investigate the feasibility of gadolinium (Gd) contrast-enhanced magnetic resonance lymphangiography (MRL) in breast cancer patients within a typical clinical setting, and to establish a Gd-MRL protocol and identify potential MRL biomarkers for differentiating metastatic from non-metastatic lymph nodes.

**Materials and Methods:**

32 patients with unilateral breast cancer were enrolled and divided into 4 groups of 8 patients. Groups I, II, and III received 1.0, 0.5, and 0.3 ml of intradermal contrast; group IV received two 0.5 ml doses of intradermal contrast. MRL images were acquired on a 3.0 T system and evaluated independently by two radiologists for the number and size of enhancing lymph nodes, lymph node contrast uptake kinetics, lymph vessel size, and contrast enhancement patterns within lymph nodes.

**Results:**

Group III patients had a statistically significant decrease in the total number of enhancing axillary lymph nodes and lymphatic vessels compared to all other groups. While group IV patients had a statistically significant faster time to reach the maximum peak enhancement over group I and II (by 3 minutes), there was no other statistically significant difference between imaging results between groups I, II, and IV. 27 out of 128 lymphatic vessels (21%) showed dilatation, and all patients with dilated lymphatic vessels were pathologically proven to have metastases. Using the pattern of enhancement defects as the sole criterion for identifying metastatic lymph nodes during Gd-MRL interpretation, and using histopathology as the gold standard, the sensitivity and specificity were estimated to be 86% and 95%, respectively.

**Conclusion:**

Gd-MRL can adequately depict the lymphatic system, can define sentinel lymph nodes, and has the potential to differentiate between metastatic and non-metastatic lymph nodes in breast cancer patients.

## Introduction

Breast cancer is the leading cause of cancer mortality in women worldwide [1]. Metastatic spread of malignancy can profoundly alter the prognosis and management of breast cancer [2]. Malignant spread to axillary lymph nodes is one of the most important predictors of survival in patients with breast cancer, increasing the 10-year recurrence rate from 20–30% to nearly 70% [2]. Since confirmation of metastatic lymph nodes alters both clinical and surgical management, and it has been well documented that post mastectomy radiation can benefit patients with axillary lymph node metastases [3].

Typically, breast cancer metastasizes to the sentinel lymph node (SLN), which is considered to be the first node to drain lymphatic fluid from the tumor [4,5]. The detection of a SLN metastasis is therefore critical for staging and prognosis. Lymphoscintigraphy, currently the most widely used methods to localize SLN, has some diagnostic and implementation disadvantages [6,7]. A radioactive tracer and associated precautions are required. Moreover, image quality and spatial resolution of scintigrams is generally poor. Intraoperative lymph node mapping with the administration of isosulfan blue (“blue dye”) is another commonly used technique to identify SLN, but is invasive and can only be performed during surgery. The possibility of tracer progression into other lymph node drainage pathways can obscure or mistakenly mimic the SLN [8]. Furthermore, while both methods can identify SLNs, neither method can differentiate metastatic from non-metastatic lymph nodes.

Magnetic Resonance Lymphangiography (MRL) is a relatively new technique consisting of the acquisition of magnetic resonance images following the interstitial injection of a contrast agent. MRL with gadolinium (Gd)-based contrast agents (Gd-MRL) can generate high spatial resolution images of lymphatic vessels and lymph nodes [9–14]. Despite substantial progress in MRL techniques [15], the single published paper on breast Gd-MRL to date used only healthy human volunteers [16]. The objective of this study is to investigate the feasibility of Gd-MRL for breast patients in a typical clinical setting, to establish an effective Gd-MRL protocol, and to identify potential MRL biomarkers for differentiating metastatic from non-metastatic lymph nodes.

## Materials and Methods

### Patients

The study was conducted after approval of the Shanghai Jiao tong University School of Medicine institutional review board, and was performed in accordance with the ethical guidelines of the Declaration of Helsinki. Written informed consent was obtained for each patient. From April 2012 to July 2012, a total of 32 patients (ages ranging from 27 years to 71 years; mean age: 51.5±9.2 years) were enrolled. Patient inclusion criteria were: (1) the presence of invasive breast cancer in a single breast, and (2) a treatment plan that included axillary lymphadenectomy. Pregnant patients, patients with previous axillary lymphadenectomy, and patients with renal insufficiency were excluded.

### Contrast agent

Gadopentate dimeglumine (Gd-DPTA) (Magnevist, Bayer Schering Pharma AG, Berlin, Germany) was mixed 10:1 with 1% mepivacaine hydrochloride, and used for all injections. Mepivacaine hydrochloride was added to alleviate pain during intradermal injection. All injections were performed using a 1 ml tuberculin syringe and a 26 G needle.

### MR Imaging

Imaging was performed on a 3.0 T MR scanner (Achieva TX, Philips Medical Systems, Best, Netherlands). Patients were placed in the prone position, head first in the scanner using a dedicated seven-channel phased-array breast coil. The imaging protocol consisted of an axial T1-weighted fast spin echo (T1-FSE) sequence, an axial diffusion-weighted imaging (DWI) echo-planar sequence (TE/TR 6548/65 ms; flip angle 90°, FOV 340×340 mm^2^, acquired voxel size 2 × 2 × 3 mm^3^; b values of 0 and 600 sec/mm^2^), and an axial and two sagittal T2-weighted fat suppressed sequence (Philips SPAIR; TE/TR 120/90 ms; inversion delay 125 ms; flip angle 90°; FOV 340× 340 mm^2^, acquired voxel size 1.01×1.31×3.0 mm^3^). For Gd-MRL, 3D fast spoiled gradient-recalled echo T1-weighted coronal images with a fat saturation (T1 high-resolution isotropic volume excitation, THRIVE) were acquired prior to the administration of Gd-DTPA with the following parameters: TR/TE: 3.5/1.7ms, flip angle: 25, FOV: 375×350mm^2^, matrix: 750×700, slices: 150, voxel size: 1.2×0.5×0.5 mm^3^, acquisition time: 3 min. After intradermal administration of the contrast material, the same imaging sequence (THRIVE) was repeated at 9, 12, 15, 18, 21 and 24 minutes. MIPs (Maximum Intensity Projections) were used to improve visualization of lymphatic vessels. Finally, dynamic fat suppressed axial high-resolution T1-weighted fast gradient echo images (THRIVE) were acquired (TE/TR 3.4/1.3 ms; flip angle of 10°; FOV 340×340 mm^2^, acquired voxel size 1×1×1.5 mm^3^); temporal resolution was 60s per dynamic acquisition, with a total of six dynamic acquisitions, one obtained prior to and five obtained immediately after intravenous administration of a bolus injection of 0.1 mmol/kg gadopentate dimeglumine followed by a 20 ml saline flush at an injection rate of 2 ml/s using an automatic injector. Total imaging time for each patient was about 45 minutes.

To optimize the breast MRL procedure, 32 patients were assigned to one of four groups (8 patients per group), with each group representing a different contrast administration or contrast dosing protocol. Both breasts were injected in each patient in all groups. For groups 1-3, gadolinium-based contrast was administered intradermally into the upper-outer periareolar area; only the amount of contrast delivered varied between the groups. Groups 1, 2, and 3 had 1.0 ml, 0.5 ml, and 0.3 ml of contrast administered, respectively. Patients in group 4 received two 0.5 ml intradermal periareolar injections with one injection in the upper-outer and one in the lower-inner quadrants. For all groups, following each injection, finger massage was applied for about 90 seconds at the injection site in order to facilitate the penetration of contrast into the lymphatic system. Table 1 summarizes the above mentioned information.

**Table 1:** Total number of patients and number of axillae imaged within each group.

**Group**	**Injection volume**	**Injection sites**	**Total number of imaged axillae**
1	1.0 ml	1	16
2	0.5 ml	1	16
3	0.3 ml	1	16
4	0.5 ml	2	16

### Image analysis

**Figure 1:**
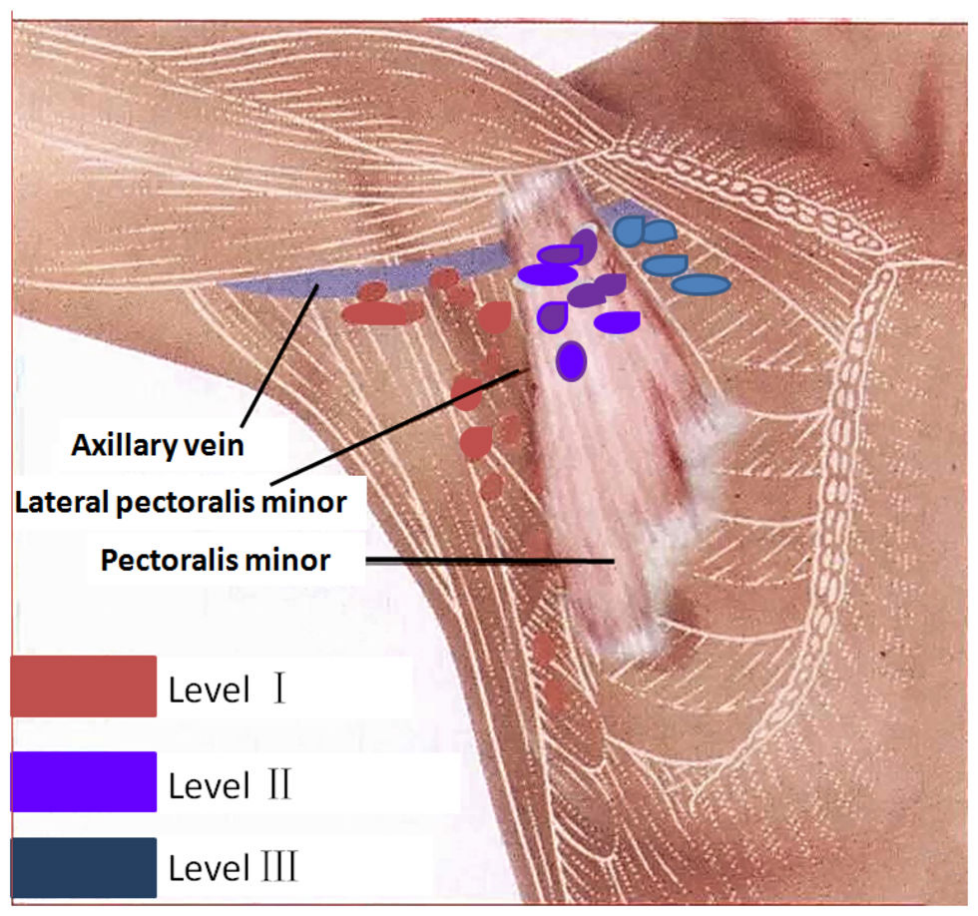
Diagrammatic representation of axillary lymph node levels. Lymph nodes were classified as Level 1, Level 2, or Level 3 based on anatomic location. Level I: latissimus dorsi to lateral pectoralis minor; level II: posterior to pectoralis minor; level III: medial pectoralis minor to thoracic inlet.

Images were evaluated by two radiologists with over 15 years of body MRI experience. Morphological characteristics, the number of enhancing axillary lymph nodes, number of enhancing breast lymphatic vessels, and the average signal intensity for each lymph node were evaluated independently using a workstation (ViewForum, release 5.1; Philips Healthcare). Morphological analysis included evaluation of contrast enhancement defects and the presence of lymphatic vessel dilation. Contrast enhancement defects are foci of nonenhancement within an enhancing lymph node, using criteria adopted from the literature [17–20]. For morphological characteristics of lymphatic vessels, we defined a lymphatic vessel as dilated if its diameter was more than 1.5 mm or at least 50% greater than the diameter of lymphatic vessels in the contralateral breast. Lymph nodes were further classified as Level 1, Level 2, or Level 3 based on anatomic location (Figure 1). The maximum transverse diameter (short axis diameter) of enhancing axillary lymph nodes and lymphatic vessels were measured and recorded. Contrast uptake kinetics for all enhancing lymph nodes were acquired from operator-defined regions of interest (ROI) drawn on both the pre- and post-contrast images. The signal intensity of air (ROI, 300 mm^2^) was used to estimate the background signal. All measurements were performed three times and each signal amplitude value was calculated as the mean of 3 separately-sampled ROIs. Time-signal intensity curves of lymph nodes were then constructed from signal intensity (SI) values obtained from the ROIs. The maximal enhancement time was defined at time point that the lymph node signal intensity began to reach plateau. The nodal signal-to-noise ratio (SNR, the maximum enhancement height) and nodal enhancement ratio (ER) at the maximal enhancement time were subsequently calculated. The nodal maximum enhancement height was determined by dividing the mean nodal signal intensity at the maximal enhancement time by the standard deviation (SD) of noise measured outside the patient. Nodal ER was determined by mean nodal SI at maximal enhancement time subtracting nodal SI at pre-contrast and divided by the nodal SI before contrast, and converted to percentages. MRL data from patients in group 1, 2, 4 were used to explore the potential of Gd-MRL for differentiating metastatic lymph nodes from non-metastatic ones.

After the MRL, a radiologist with 22 years of breast MRI experience and a surgeon with 15 years of breast surgery experience worked together to correlate the MR images with dissected lymph nodes based on location, size, morphological characteristics, and the number of metastatic lymph nodes in the patient by pathological evaluation. A pathologist with 8 years of experience who was blinded to the MRL results performed pathological evaluations using standard departmental procedure [21].

### Statistical Analysis

The number of lymph nodes, SNR, ER, and number of lymphatic vessels were compared between the four groups. The ANOVA test was used to determine the statistical significance of the different results among the four groups. Inter-observer agreement was calculated by using the kappa test, with *k* values of 0.5-0.75 considered to indicate satisfactory agreement, and values higher than 0.75 were considered to indicate excellent agreement. A *p* value of < 0.05 was used as the cutoff for statistical significance.

## Results

All breast cancer patients completed their examinations successfully without any unexpected adverse events. A small papular eruption typically appeared in the periareolar area at the site of contrast injection, and was most obvious about 30 minutes following the injection. All side effects are summarized in Table 2.

**Table 2:** Clinically observed side effects for all patients in group 1-4.

**Group**	**Diameter of local redness and swelling (cm)**	**Subsiding time of local redness and swelling (hours)**	**Cases of local blister appearing**	**Local infection**
1	2.7	16.9	3	None
2	1.6	8.7	None	None
3	1.2	5.7	None	None
4	1.7	9.6	None	None

**Figure 2:**
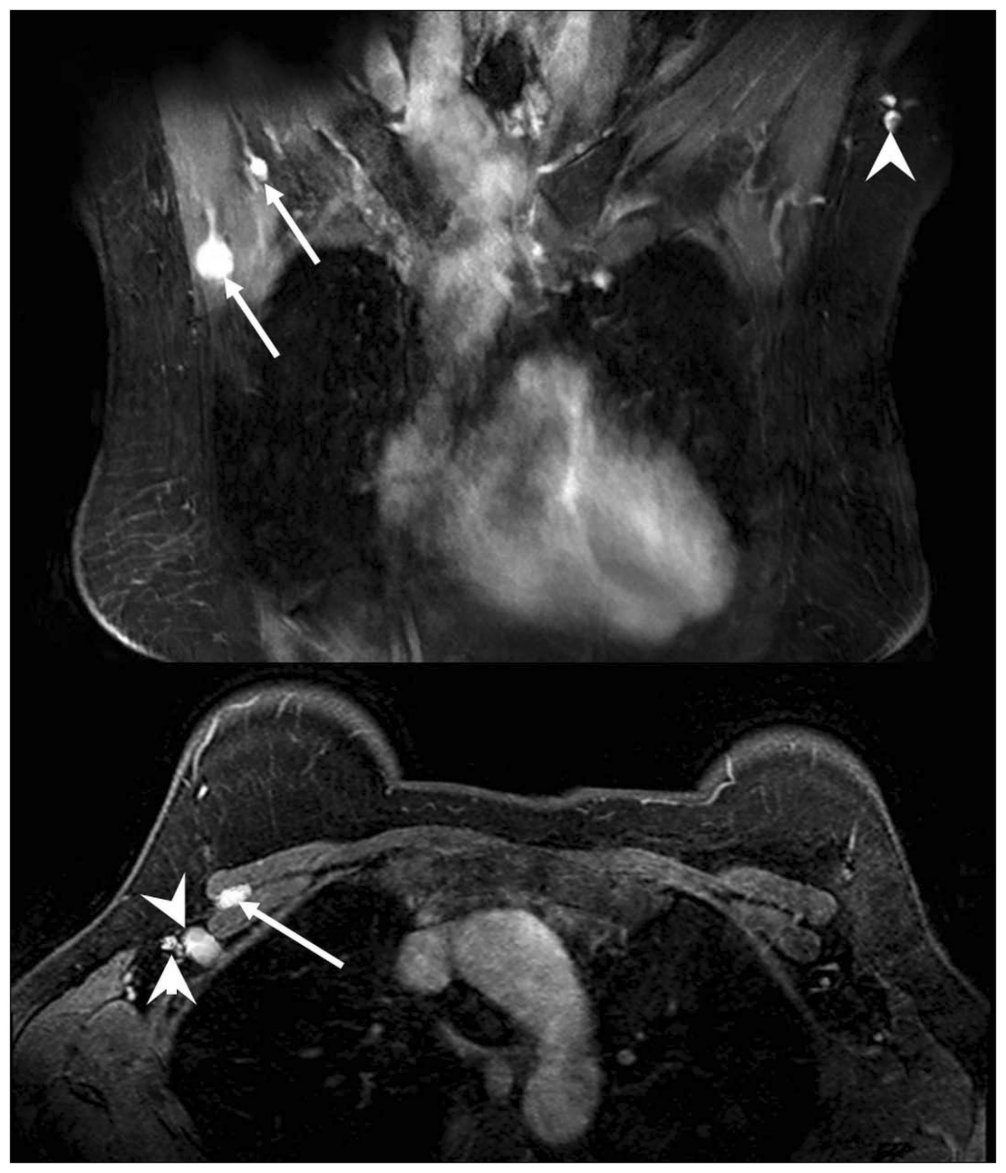
MRL images of breast lymph nodes at different levels. (A) (coronal plane) and (B) (transversal plane) show typical MRL images illustrating breast lymph node enhancement at different nodal levels. *Arrowhead*: Level 1 axillary lymph nodes. *Arrows*: Level 2 axillary lymph nodes.

**Figure 3:**
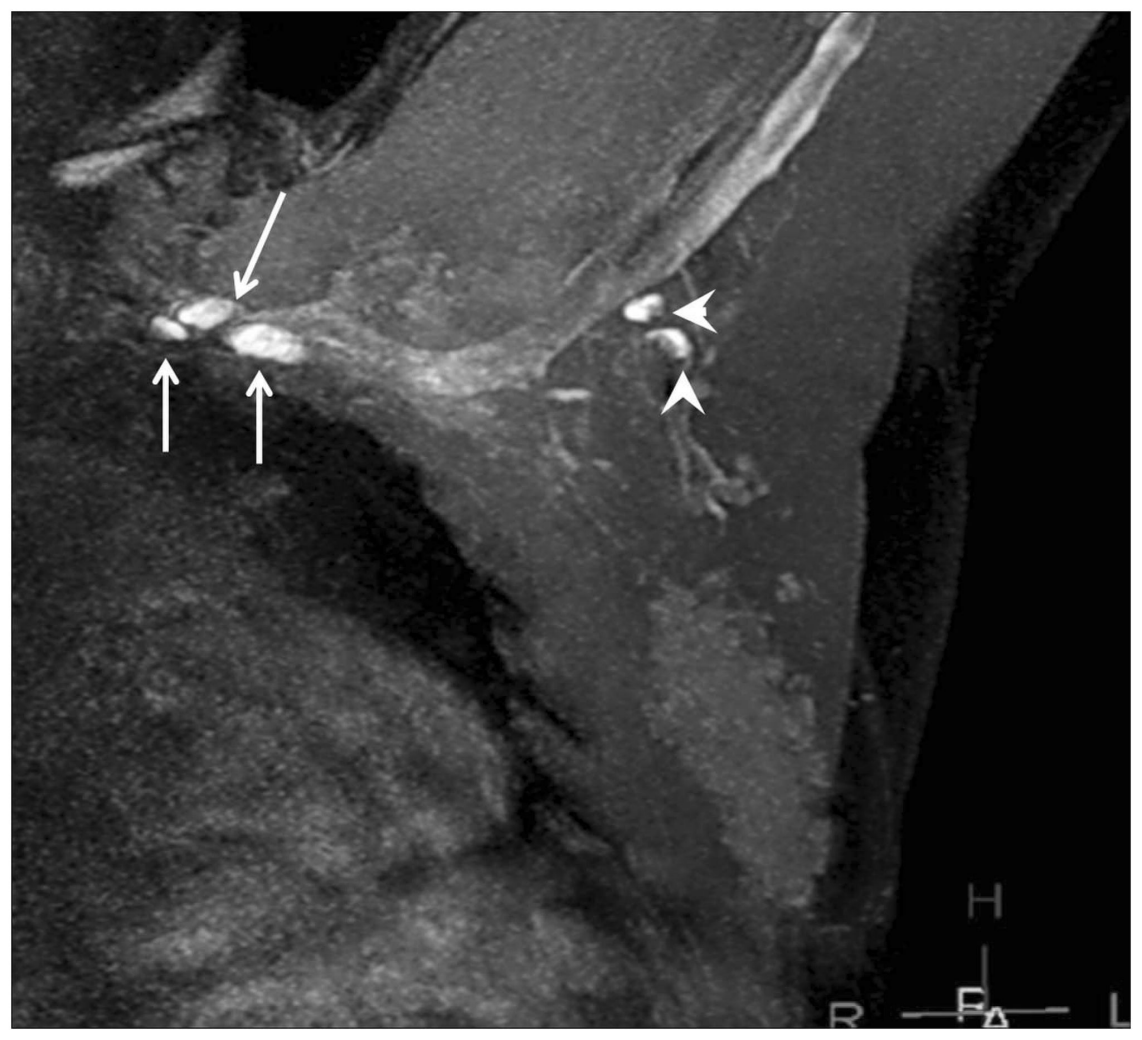
MRL images of breast lymph nodes at different levels. A typical MRL image shows enhancing breast lymph nodes at different nodal levels. *Arrowhead*: Level 1 lymph nodes. *Arrow*: Level 3 lymph nodes.

Evaluation of lymph node and lymphatic vessel, including the number, diameter, and signal intensity showed excellent inter-observer agreement, with *k* values of 0.97, 0.88, and 0.96, respectively. Typical examples of enhancing Level 1 through Level 3 axillary lymph nodes on MRL are shown in Figures 2 and 3. The overall number and diameters of enhancing lymph nodes in each of the four groups are summarized in Table 3.

**Table 3:** Comparison of number and diameter of lymph nodes and lymph vessels in group 1-4.

		**Enhancing axillary lymph nodes**					**Lymphatic vessels in breast**	
**Group**	**Axillas (N)**	**Number (Median)**				**Diameter (mm)**	**Number**	**Diameter (mm)**
		**Level 1**	**Level 2**	**Level 3**	**Total number**	**Mean±SD**	**Median**	**Mean±SD**
1	16	3.5	1	0.5	5	9.13 ± 2.29	3	1.42 ± 0.53
2	16	3.5	1	0.5	4.5	8.57 ± 1.97	3	1.39 ± 0.53
3	16	1.5^*^	0	0	2^*^	8.21 ± 1.67	1^*^	1.34 ± 0.53
4	16	4	1	0.5	5.5	8.24 ± 2.19	3	1.62 ± 0.46

Note: * indicates a significant difference between group 3 and the other three groups

Groups 1, 2, and 4 had a significantly greater number of enhancing lymph nodes than group 3 (F ratio = 26.41, p<0.001), but otherwise there was no significant difference between groups 1, 2 and 4. There were a significantly greater number of enhancing Level 1 lymph nodes in groups 1, 2, and 4 compared to group 3 (F ratio = 15.34, p<0.001), but no significant differences in the number of enhancing Level 2 or Level 3 lymph nodes between any of the four groups, and no significant difference in the number of enhancing Level 1 lymph nodes between groups 1, 2, and 4. Similarly, the number of enhancing lymphatic vessels visualized in groups 1, 2, and 4 was significantly greater than those of group 3 (F ratio = 9.643, p<0.001), but there was no significant difference in the number of enhancing lymph vessels between groups 1, 2, and 4.

**Figure 4:**
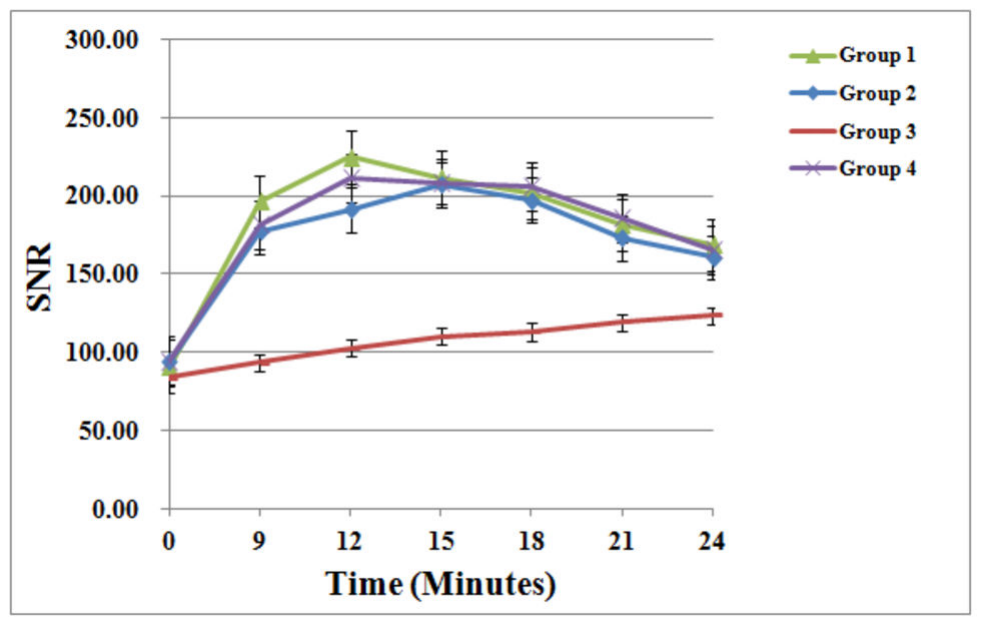
Axillary lymph nodes contrast-uptake kinetics of the four patient groups. Graph of enhancement profile over time as determined with signal-to-noise ratio (SNR) measurements in the axillary lymph node shows a different contrast -uptake kinetics of the four patient groups. ANOVA test results show that the SNR (at the maximum enhancement height) of the lymph nodes in group 1, 2 and 4 was significantly higher than that in group 3 (SNR: F ratio=17.613, p<0.001), though there was no significant difference between groups 1, 2 and 4. Error bars represent standard errors of the mean.

Figure 4 shows contrast uptake curves for all groups, and Table 4 summarizes the time needed for axillary lymph nodes to reach peak enhancement and the enhancement rate (ER). ANOVA results show that the SNR and ER of the lymph nodes in group 1, 2 and 4 were significantly higher than that in group 3 (SNR: F ratio=17.613, p<0.001; ER: F ratio=8.721, p<0.001), though there was no significant difference between groups 1, 2 and 4.

**Table 4:** Differences in dynamic contrast-enhanced kinetics between groups 1-4.

**Group**	**Time to maximal enhancement (minutes)**	**Axially lymph node at maximal enhancement time（Mean±SD)**	
		**SNR**	**ER (%)**
1	12	211.23 ± 53.07	145.91 ± 69.15
2	15	202.67 ± 22.09	124.19 ± 31.16
3	24	124.63 ± 12.81^*^	51.57 ± 27.99^*^
4	12	217.63 ± 41.53	129.19 ± 49.78

Note: * indicates a significant difference between group 3 and the other three groups

**Figure 5:**
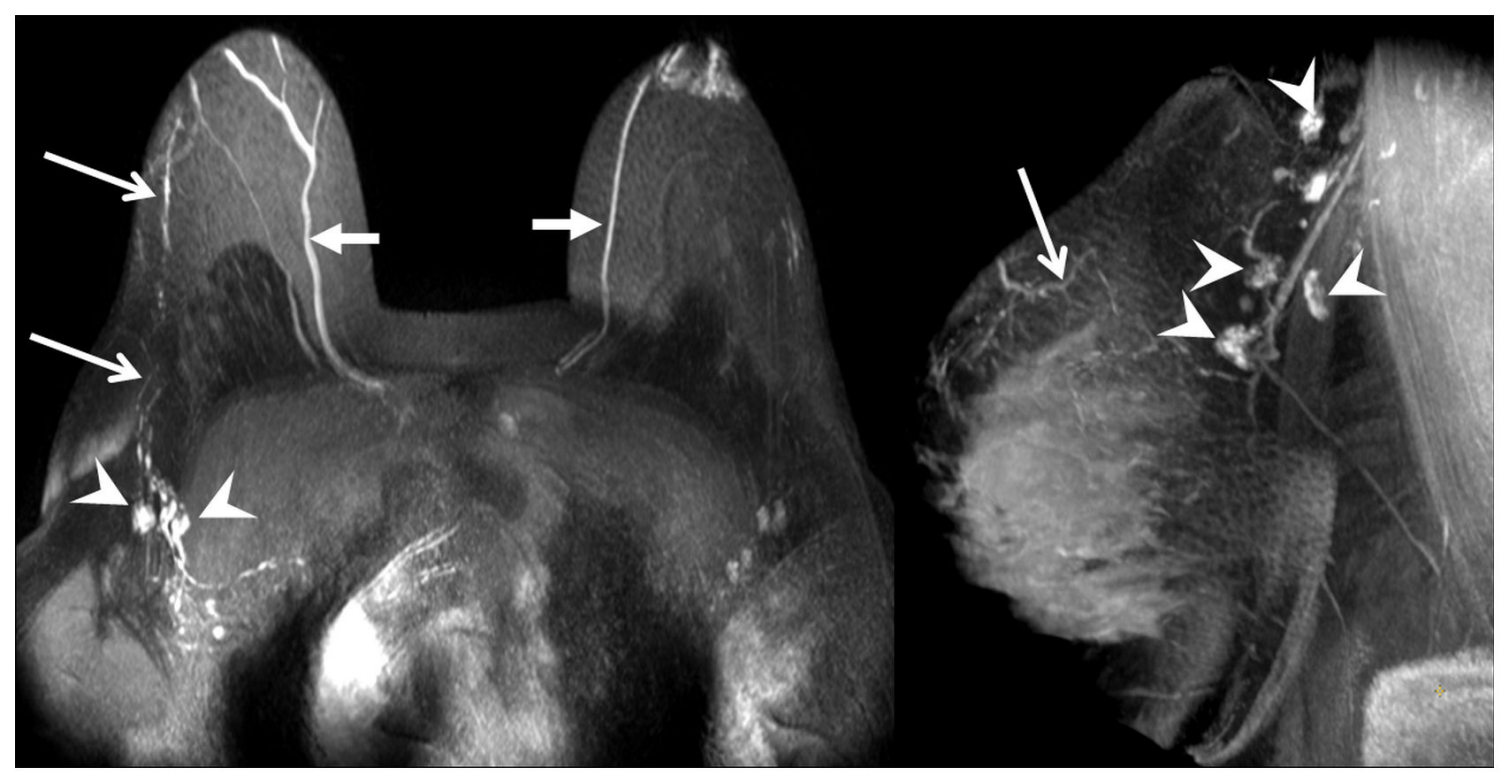
MRL images of breast normally-sized metastatic lymph nodes. Example of normally-sized metastatic lymph nodes with an abnormal nodal enhancement pattern and lymphatic vasculature. *Arrowheads*: enhancement defects within non-enlarged lymph nodes. *Long arrows*: lymphatic vessels. *Short arrows*: veins.

**Figure 6:**
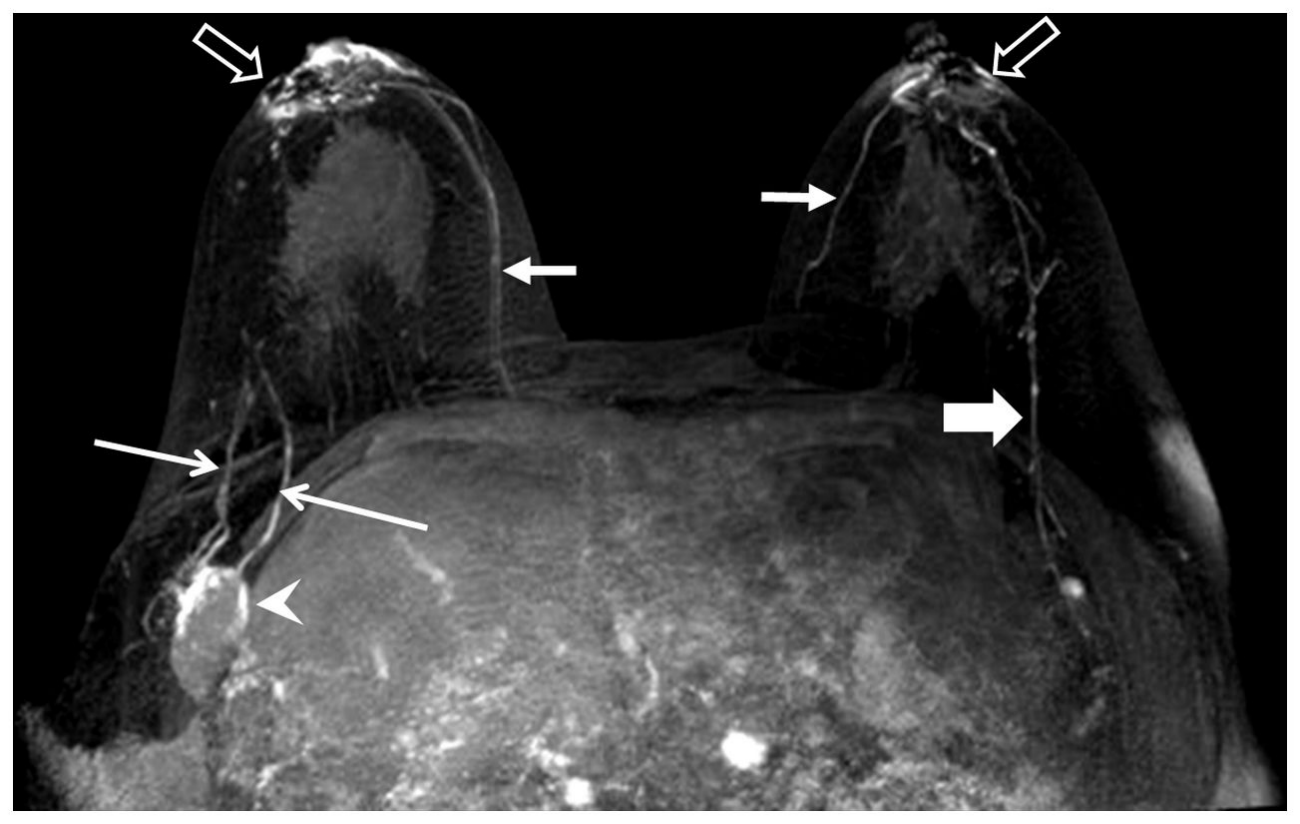
MRL of enlarged metastatic lymph nodes. Arrowhead: Irregular enlarged lymph nodes with enhancement (or contrast filling) defect pattern. *Long thin arrows*: enhancing and dilated lymphatic vessels. Short fat arrow: enhancing normal lymphatic vessels with a beaded appearance. Short arrows: vein. Hollow arrows: injection sites.

Table 5 lists the results for MRL morphological characteristics of SLNs from patients in groups 1, 2 and 4. Using histopathology as the gold standard, the presence of contrast enhancement defects gave a sensitivity of 86% and specificity of 95%. In addition to SLN morphological analysis, 27 of 128 (21%) lymphatic vessels were found to be dilated. All patients with dilated lymphatic vessels were pathologically proven to have metastases. Figure 5 is a representative MRL for a patient with a normal-size metastatic lymph node, showing an enhancement defect pattern and a dilated lymphatic vessel. Figure 6 is another example, but with an enlarged metastatic lymph node. Excepting size, it has the same morphological characteristics as the lymph node in Figure 5.

**Table 5:** Summary of lymph node analysis for patients in group 1, 2 and 4.

**MRL findings**	**Number of lymph nodes with contrast enhancement defects**		**Number of lymph nodes without contrast enhancement defects**			
	**Number of non-Metastatic lymph nodes**	**Number of Metastatic lymph nodes**	**Number of non-Metastatic lymph nodes**	**Number of Metastatic lymph nodes**	**Sensitivity**	**Specificity**
Group 1	2	6	51	1		
Group 2	1	10	58	2	86.2%	95.3%
Group 4	2	9	55	1		

## Discussion

Previous human studies have demonstrated that Gd-MRL is a safe method for imaging lymphatic system, and can be used to delineate the anatomy of lymphatic vasculature in various parts of the body [9,13,22] including the breast [16]. The sub-areolar plexus (Sappey’s plexus) is known to have a rich lymphatic network. Periareolar intradermal injection of contrast agent was chosen for this study based on the consensus that it is an end point for dermal lymphatic drainage, and subsequently drains to the axilla [23,24]. Gadopentate dimeglumine is a commercially available, widely used extracellular, water-soluble paramagnetic contrast agent. The recommend dose for intravenous administration is 0.1 mmol per kilogram of body weight, which is about 5 times higher than the concentration used in this study. A favorable safety profile has been previously shown for intravenous administration [25]. Although the smaller dose and local injection into the breast lowers the risk of anaphylactoid reactions and renal toxicity, we excluded patients with renal failure as a general precaution. Animal studies indicate that gadopentetate dimeglumine causes moderate necrosis, hemorrhage, inflammation and edema that is not statistically different from meglumine diatrizoate when the agent is injected into the subcutaneous tissues of rats or mice [26,27]. Periareolar intradermal contrast injections can cause minor tissue injury, namely superficial erythema and blistering at the injection site. We found that tissue injury at the injection sites was temporary and reactions fell within the expectations of clinical experience [28] Moreover, no irreversible or unexpected adverse events have been reported in prior contrast-enhanced MRL studies, which is confirmed by our observations [10,12,13,29,30].

Comparison of results from the four groups suggest that a single (per breast), a 0.5 ml periareolar gadolinium injection is sufficient for imaging axillary lymph nodes. More specifically, for axillary lymph nodes, the maximum peak enhancement from a 0.5 ml gadolinium contrast injection is comparable to a 1.0 ml injection, but with fewer side effects. Conversely, insufficient enhancement was seen with the administration of 0.3 ml of contrast. The protocol for group 4, (two 0.5 ml periareolar injections) was not found to be superior to the single injection of 1.0 or 0.5 ml gadolinium contrast agent, though this method could be helpful during training to ensure an intradermal rather than a subdermal (subcutaneous) injection. The only statistically significant advantage for using the protocol 1 or protocol 4 over protocol 2 was about a 3 minute faster time to reach maximum peak enhancement in axillary lymph nodes, which could be valuable in clinical settings where an abbreviated waiting period between intradermal injection and MRL data acquisition is required.

The importance of identifying SLN metastases in breast cancer patients has long been recognized. The physiology of Gd-MRL makes the identification of SLNs readily apparent due to gadolinium-based contrast uptake within lymphatic vessels and lymph nodes following intradermal injection. As indicated in Table 5 and in Figures 5 and 6, Gd-MRL cannot only identify SLN, but also has the potential to differentiate metastatic from non-metastatic lymph nodes. Specifically, in addition to the well-known parameter of enlarged short axis dimension of lymph nodes, contrast enhancement defects could be an additional biomarker identifying metastatic lymph nodes. The observation of contrast enhancement defects within metastatic lymph nodes is in agreement with previous MRL studies on cervical lymph node metastases in a rabbit model [31], and malignant lymphoma in a mouse model [32]. Our results also suggest that lymphatic vessels that connect to metastatic lymph nodes can appear dilated compared to non-metastatic vessels, an observation consistent with previous studies using CT lymphography [33]. To our knowledge, the morphological features of lymph node enhancement defects and dilated lymphatic vessels have not been previously observed in breast cancer patients, and more importantly, these features show potential for differentiating metastatic from non-metastatic lymph nodes. The mechanism which underlies these observations is not fully known. Elucidating the mechanism requires further thorough scientific investigation and is beyond the scope of this study. Nevertheless, we can speculate based on our observations and related knowledge in the literature. In the normal lymphatic system, intradermal contrast material diffuses into lymphatic vessels and drains with lymph fluid through the lymphatic system to lymph nodes. In metastatic lymphatic systems, however, lymphatic vessels can be partially blocked by metastatic implants (perhaps dilating lymphatic vessels) or be completely absent due to metastatic invasion. This can cause the enhancement defects on MRL images as observed in this study. Finally, our results are also in agreement with the well-documented knowledge that metastatic lymph nodes can be enlarged [34–39].

This study has several limitations. First, there are technical challenges preventing precise node-by-node correlation, which is well-understood by the community. What we did was to correlate MR results with pathologic results based on 1) the size and location of the lymph node, 2) the number of metastatic lymph nodes as determined by pathological examination and 3) corresponding number of lymph nodes with the most suspicious MRL patterns. That is, only if the number of metastatic lymph nodes by pathological examination was not equal to the number of lymph nodes with Gd-MRL enhancement defects or dilated lymphatic vessels, did we consider the results to be a mismatch between MRL and pathology. Second, the 32 (8 patients in 4 groups) patients had four different MRL protocols. Although these protocol differences did not affect our ability to identify potential MRL biomarkers for differentiating metastatic from non-metastatic lymph nodes, they do affect the accuracy of our statistical results. We are actively seeking a better way to improve the correlation between MRI results and pathologic finding. We are also actively recruiting more patients in an effort to overcome the above limitations. Finally, we only evaluated the intradermal periareolar injection. Other possible injection sites include subareolar, subcutaneous over the primary tumor site, peritumoral, and intratumoral. As mentioned before, Gd-MRL for patients with breast cancer is an essentially unexplored area, and further thorough investigations would have to be performed prior to routine clinical use.

In conclusion, we have successfully acquired MRL images in breast cancer patients using a widely available Gd-based contrast agent in a typical clinical setting. Our results demonstrate the potential of Gd-MRL to identify sentinel lymph nodes and differentiate metastatic from non-metastatic lymph nodes.
